# Asian flush gene variant increases mild cognitive impairment risk: a cross-sectional study of the Yoshinogari Brain MRI Checkup Cohort

**DOI:** 10.1265/ehpm.24-00214

**Published:** 2024-10-11

**Authors:** Mikiko Tokiya, Manabu Hashimoto, Kenji Fukuda, Kazuhiro Kawamoto, Chiho Akao, Mariko Tsuji, Yusuke Yakushiji, Haruki Koike, Akiko Matsumoto

**Affiliations:** 1 Department of Environmental Medicine, Saga University, 5-1-1 Nabeshima, Saga 849-8501, Japan; 2 National Hospital Organization Hizen Psychiatric Medical Center, 160 Mitsu, Yoshinogari-machi, Kanzaki-gun, Saga 842-0192, Japan; 3 Department of Cerebrovascular Disease, St. Mary’s Hospital, 422 Tsubukuhonmachi, Kurume, Fukuoka 830-8543, Japan; 4 Department of Neurology, Kansai Medical University, 2-5-1 Shinmachi, Hirakata, Osaka-fu 573-1010, Japan; 5 Division of Neurology, Department of Internal Medicine, Faculty of Medicine, Saga University, 5-1-1 Nabeshima, Saga 849-8501, Japan

**Keywords:** Aldehyde dehydrogenase 2, Dementia, Mild cognitive impairment (MCI), Prevention, Race, rs671

## Abstract

**Background:**

The East Asian-specific genetic diversity, the rs671 variant of aldehyde dehydrogenase 2, causes the “Asian flush” phenomenon following alcohol consumption, resulting in an alcohol avoidance phenotype. The variant is suggested as a risk factor for Alzheimer’s disease; however, its association with mild cognitive impairment (MCI), an effective target for secondary prevention of dementia, remains unclear.

**Method:**

This cross-sectional study examined 430 individuals aged 60–80 years (251 women) without overt cognitive impairment in Yoshinogari, Japan. The effect of the rs671 variant on MCI, defined by scores <26 or <25 on the Japanese version of the Montreal Cognitive Assessment, was evaluated using multivariate logistic regression.

**Results:**

The models included *APOEε4*, sex, age, education, history of habitual drinking, Brinkman index, hypertension, diabetes, and subclinical magnetic resonance imaging findings and consistently estimated the risk of the rs671 variant. Subsequently, stratified analyses by history of habitual drinking were performed based on an interactive effect between rs671 and alcohol consumption, and the rs671 variant significantly influenced MCI in participants who did not drink habitually, with odds ratios ranging from 1.9 to 2.1 before and after adjusting for covariates, suggesting an association independent of hippocampal atrophy and small vessel dysfunction. Conversely, no such association with the rs671 variant was observed in participants with a history of habitual alcohol use. Instead, hippocampal atrophy and silent infarcts were associated with MCI.

**Conclusions:**

This is the first study to demonstrate an association between the rs671 variant and MCI morbidity. The findings highlight the need for race-specific preventive strategies and suggest potential unrecognized mechanisms in dementia development.

**Supplementary information:**

The online version contains supplementary material available at https://doi.org/10.1265/ehpm.24-00214.

## 1. Introduction

Dementia is a clinical syndrome characterized by a decline from a previously attained level of cognition that affects an individual’s activities of daily living and social functioning [1]. Globally, the prevalence of dementia was 47 million people in 2015 and is expected to increase to 131 million by 2050 [1]. Recognizing this growing concern, the World Health Organization’s Global Action Plan has designated dementia as a global priority [2]. Mild cognitive impairment (MCI) is considered a precursor or high-risk state of dementia [3] and is regarded as an important concept in dementia prevention.

The rs671 genetic variant of the *aldehyde dehydrogenase 2* gene (*ALDH2*) is specifically prevalent in East Asian countries such as Japan, China, South Korea, and Taiwan [4–7], leading to region-specific disease structures [8–10]. This variant causes the “Asian flush” phenomenon due to a disturbed function resulting from the alteration of the 3-dimensional structure of the ALDH2 dimer [11]. When rs671 variant carriers consume alcohol, acetaldehyde, a substrate of ALDH2, accumulates in the blood, causing skin flushing, nausea, palpitations, drowsiness, and headache [8, 12, 13]. This reaction is believed to be the primary reason for lower alcohol consumption and frequency among rs671 variant carriers [14]. The essential role of ALDH2 is the metabolism of endogenous aldehydes, not just acetaldehyde, as evidenced by ALDH2 expression in various species, including algae, fish, rodents, and primates [15]. Formaldehyde and 4-hydroxynonenal, substrates of ALDH2 [16–18], are produced endogenously and are closely related to cognitive function [19–21].

In animal models with low ALDH2, similar to rs671 carriers, Alzheimer’s disease-like pathological findings are observed, including cognitive decline, increases in amyloid protein, and tau protein phosphorylation [19, 22–24]. However, epidemiological evidence is limited. A case-control study has suggested an association between rs671 and Alzheimer’s disease [25, 26], noting a synergistic effect of the rs671 variant and *APOEε4*, a missense variant of the apolipoprotein E gene, an established risk factor for Alzheimer’s disease [25]. The Mini-Mental State Examination, the most popular tool for distinguishing cognitive impairment, revealed an association between the rs671 variant and cognitive impairment in Chinese oldest-olds [27]. However, MCI, an earlier and more suitable stage for intervention, has not been investigated. The pathological roles of alcohol consumption, hippocampal atrophy, and small vessel dysfunction are also of great interest.

To address this gap, we conducted a cross-sectional study to examine the association between *ALDH2* rs671 and MCI in Japanese residents using the database of health checkups, including brain magnetic resonance imaging (MRI), performed by the Psychiatric Medical Center as a contribution to the local community.

## 2. Methods

### 2.1. Participants

The participants were residents of the Yoshinogari area, Sefuri-cho, Kanzaki-city, Saga Prefecture, aged 60 years or above, who were independent in their daily activities and had no apparent cognitive impairment, and who voluntarily participated in the Brain MRI Checkup. The National Hospital Organization Hizen Psychiatric Medical Center has been conducting the checkups since 1997 as a community benefit activity [28], and data obtained from 2018 to 2021 were used for this study. Among 455 participants, 25 individuals were excluded based on the following criteria: dental problems, claustrophobia, or contraindications to MRI (6); dialysis patients (1); brain tumors (including suspected) (4); autoimmune disorders (collagen disease, ulcerative colitis) (2); cerebrovascular disease (postoperative cerebral aneurysm, previous cerebral infarction, subarachnoid hemorrhage, stroke) (5); other cerebral neurological diseases (anterior brain cyst, REM sleep disorder, cerebral atrophy, progressive supranuclear palsy) (4); depression (1); cardiac disease (1); and many omissions in the questionnaire (1).

This study was conducted with the approval of the Ethics Commission of the National Hospital Organization Hizen Psychiatric Medical Center (No. 24-4, 30-8, 30-38) and in accordance with the Declaration of Helsinki.

### 2.2. Self-administered questionnaire

The questionnaire collected data on years of education, drinking status, and smoking status, which are factors potentially affecting cognitive function and rs671 [10, 14, 29–31]. Education levels were categorized as follows: completion of compulsory education (≥9, <12 years), high school graduation (≥12, <14 years), completion of junior college or professional training college (≥14, <16 years), and university or graduate school completion (≥16 years). Years of habitual drinking were classified into seven categories: 0 = “never,” 1 = “<10 years,” 2 = “10–19 years,” 3 = “20–29 years,” 4 = “30–39 years,” 5 = “40–49 years,” and 6 = “≥50 years.” Participants also reported their current alcohol consumption. The Brinkman index was calculated from the number of cigarettes smoked per day and the number of years of smoking.

### 2.3. Genotyping

On the examination day, DNA samples were collected by swabbing the participants’ inner cheeks with sterile cotton swabs. The cotton swabs were soaked in distilled water, centrifuged to precipitate cell components, and the supernatant was removed. The cell components were incubated with Direct PCR Lysis Reagent (VIAGEN Biotech, Los Angeles, CA) and proteinase K at 55 °C overnight, followed by incubation at 85°C for 45 min to be used as PCR templates. TaqMan^®^ SNP Genotyping Assays (Thermo Fisher Scientific, Inc., Waltham, MA, USA) were performed with mixtures of primers and probes (C_11703892_10, C_3084793_20, C_904973_10) for *ALDH2* rs671 (G > A), *APOE* rs429358 (T > C), and rs7412 (C > T) according to the manufacturer’s protocol. The *APOE* genotype was determined by the combination of rs429358 and rs7412 (Table S1). In cases with rs429358 T/C and rs7412 C/T, *APOEε1ε3* and *APOEε2ε4* were indistinguishable (N = 6). Additionally, real-time PCR failures occurred in 6 samples due to issues with either rs429358 or rs7412.

### 2.4. Physical measurement and serological tests

Blood pressure was measured by medical doctors using a mercury sphygmomanometer. Peripheral blood was collected to determine levels of blood glucose, glycated hemoglobin (HbA1c), low-density lipoprotein (LDL)-cholesterol, triglycerides (TG), and high-density lipoprotein (HDL)-cholesterol. Hypertension was defined as systolic blood pressure ≥ 140 mmHg, diastolic blood pressure ≥ 90 mmHg, or current treatment for hypertension. Diabetes was defined as fasting blood glucose ≥ 126 mg/dL, blood glucose ≥ 200 mg/dL, HbA1c ≥ 6.5%, or current treatment for diabetes. Dyslipidemia was defined as LDL-cholesterol ≥ 140 mg/dL, TG ≥ 150 mg/dL, HDL-cholesterol < 40 mg/dL, or current treatment for dyslipidemia.

### 2.5. Neuroendoscopic assessment

#### 2.5.1. Japanese version of the Montreal Cognitive Assessment (MoCA-J)

The Japanese version of the Montreal Cognitive Assessment (MoCA-J) [32, 33] was used to assess cognitive function. MoCA-J is a cognitive screening test designed for the early detection of cognitive impairment. A score of <26 was used as the cutoff value for mild cognitive impairment (MCI), with an expected sensitivity of 70–100% and specificity of 50–87% [33–35]. MoCA-J assessments were conducted by nurses or psychologists trained by neurologists in accordance with MoCA-J procedures [32].

#### 2.5.2. MRI

MRI scans were performed by skilled technicians at the National Hospital Organization Hizen Psychiatric Medical Center. Hippocampal and surrounding area atrophy (VSRAD-atrophy) was evaluated using the Voxel-Based Specific Regional Analysis System for Atrophy Detection (VSRAD) advance version^®^, which employs Statistical Parametric Mapping 8 for accurate segmentation [36]. The gray matter volumetric density within each voxel of the parahippocampal gyrus, amygdala, hippocampus, and dorsal brainstem was measured to calculate a Z-score: ([health control mean] − [individual value])/(health control S.D.). The total Z-score for voxels showing atrophy (positive Z-score) was divided by the number of these voxels to yield the VSRAD-atrophy score: (VSRAD-atrophy = [Total of positive Z-score]/[Number of positive voxels]). A VSRAD-atrophy score of 1 or above indicates atrophy exceeding age-related changes [36].

Two neurologists further evaluated small vessel-related subclinical findings. Disagreements were resolved through consensus. Silent brain infarction (SBI) was defined as areas ≥3 mm in diameter with low signal intensity on T1-weighted images and high signal intensity on T2-weighted images [37]. Microbleeds were identified as rounded areas with homogeneous signal loss less than 10 mm in diameter on susceptibility-weighted images [38]. Age-related cerebral white matter lesions, including deep and subcortical white matter hyperintensity (DSWMH) and periventricular hyperintensity, were assessed using the Fazekas Scale [39]. The scale grades the amount of white matter T2 high signal lesions as absent (0 points), punctate foci (1 point), beginning confluence of foci (2 points), and large fused areas (3 points), based on lesion size and confluence [39].

### 2.6. Statistical analysis

Statistical analysis was performed using SAS version 9.4 software (SAS Institute Inc., Cary, NC, USA), with the significance level set at P < 0.05. Fisher’s exact test was used to detect distribution bias in categorical variables. The Kruskal-Wallis test was applied for multigroup comparisons of age and the Brinkman index. Spearman’s rank correlation coefficient (ρ) was used to examine the correlations of rs671 variant number (0–2) with MoCA-J scores (0–30), MCI (0, 1), and alcohol-related variables (years of habitual drinking, current alcohol consumption, and drinking index).

#### 2.6.1. Dependent and independent variables

The dependent variable was the MoCA-J score. The main explanatory variable was the rs671 variant. Secondary explanatory variables included *APOEε4*, years of habitual drinking, and VSRAD-atrophy. Log transformation was applied to VSRAD-atrophy to approximate a normal distribution (skewness reduced from 1.64 to 0.28) (Fig. S1).

#### 2.6.2. Logistic regression analysis

The association between MCI (defined by primary and secondary cutoffs of MoCA-J <26 and <25, respectively) and the rs671 variant was estimated using univariate and multivariate logistic regression models. The multivariate models included genetic factors, personal characteristics, lifestyle factors, and MRI findings. Odds ratios were visualized using these models, with modifications in the categorizations of variables as detailed in the figure legends.

#### 2.6.3. Sensitivity analysis

Due to the burden on participants, we only surveyed years of habitual drinking and current alcohol consumption, leaving lifetime alcohol consumption unquantified. Given the association between alcohol consumption, dementia [30], and the rs671 variant [10, 29], exposure magnitude was considered in statistical models. In the sensitivity analysis, years of habitual drinking used in the main analysis were replaced with current alcohol consumption or the ‘drinking index.’ The ‘drinking index’ was calculated as follows:

$$\begin{array}{rl} & \text{Drinking index}\\ & \;=\text{amount of current alcohol consumption*}\\ & \;\times \text{categorical years of habitual drinking}\\ & \text{*in 10 g ethanol/day/60 kg bodyweight} \end{array}$$

where:

Categorical years of habitual drinking: 0 = “never,” 1 = “<10 y,” 2 = “10–19 y,” 3 = “20–29 y,” 4 = “30–39 y,” 5 = “40–49 y,” and 6 = “≥50 y.”

Another sensitivity analysis used a lower MoCA-J cutoff of <25, which was reported elsewhere to have an MCI sensitivity of 80.5% and specificity of 81.2% [40].

## 3. Results

### 3.1. Gene frequency

As shown in Table 1, 168 (39.1%) participants had a single rs671 variant allele (ALDH2*1/*2), and 33 (7.7%) had double variant alleles (ALDH2*2/*2), resulting in a variant allele frequency of 46.7%. These frequencies are consistent with those expected by Hardy-Weinberg Equilibrium (39.6% and 7.4%, respectively; χ^2^ = 0.001, P = 1.00). For *APOEε4*, 341 (79.3%) participants had no ε4 allele (*APOEε2/ε2*, *APOEε2/ε3*, or *APOEε3/ε3*), 73 (17.0%) had one ε4 allele (*APOEε2/ε4* or *APOEε3/ε4*), and four (0.93%) had two ε4 alleles (*APOEε4/ε4*). *APOE* genotype was undetermined in 12 (2.8%) participants. These frequencies are also consistent with Hardy-Weinberg Equilibrium expectations (81.5%, 17.5%, and 0.94%; χ^2^ < 0.001, P = 1.00).

**Table 1:** Characteristics of participants.

***ALDH2* genotype** (Number of ALDH2*2)	ALDH2*1/*1 **(0)**	ALDH2*1/*2 **(1)**	ALDH2*2/*2 **(2)**	***p-value***
**N**	229	168	33	
**Female**, **N (%)**	144 (63%)	96 (57%)	15 (45%)	0.130^a^
**Age**				
**Median (IQR)**	70 (66–74)	70 (67–74)	69 (67–77)	0.766^b^
**N (%)**				0.365^a^
60th	109 (48%)	71 (42%)	17 (52%)	
70th	118 (52%)	96 (57%)	15 (45%)	
80th	2 (0.9%)	1 (0.6%)	1 (3%)	
**Years of education, N (%)**				0.751^a^
≥9, <12	30 (13%)	18 (11%)	2 (6%)	
≥12, <14	141 (62%)	100 (60%)	19 (58%)	
≥14, <16	27 (12%)	24 (14%)	7 (21%)	
≥16	31 (14%)	26 (15%)	5 (15%)	
**Drinking status**				
**Years of habitual drinking, N (%)**				<0.001^c^
Never	117 (51%)	107 (64%)	26 (79%)	
<10 years	9 (4%)	3 (2%)	1 (3%)	
≥10, <20	19 (8%)	7 (4%)	1 (3%)	
≥20, <30	15 (7%)	15 (9%)	1 (3%)	
≥30, <40	26 (11%)	17 (10%)	2 (6%)	
≥40, <50	23 (10%)	10 (6%)	1 (3%)	
≥50	17 (7%)	7 (4%)	1 (3%)	
Unknown*	3 (1%)	2 (1%)	0 (0%)	
**Current alcohol consumption** **(g/day/60 kg body weight)**				0.001^a^
0	119 (52%)	111 (66%)	26 (79%)	
<10	37 (16%)	29 (17%)	4 (12%)	
≥10, <20	20 (9%)	12 (7%)	2 (6%)	
≥20	53 (23%)	16 (10%)	1 (3%)	
**Median (IQR)**				
0	0 (0–0)	0 (0–0)	0 (0–0)	
<10	4 (1.8–6.9)	5.1 (1.5–5.5)	3.7 (2.0–6.3)	
≥10, <20	14.8 (14.0–18.1)	12.9 (11.2–15.6)	10.1 (10.1–10.2)	
≥20	37.3 (28.1–49.9)	36.2 (26.0–49.4)	36.3 (36.3–36.3)	
**Drinking index** **(g/day × categorical years)**				0.009^a^
0	129 (57%)	116 (70%)	27 (82%)	
>0, <4	6 (3%)	4 (3%)	1 (3%)	
≥4	91 (40%)	46 (28%)	5 (15%)	
**Brinkman index**				
**Median (IQR)**	0 (0–228)	0 (0–245)	0 (0–294)	0.652^b^
**N (%)**				0.251^a^
0	153 (67%)	113 (67%)	19 (58%)	
>0, <240	18 (8%)	12 (7%)	5 (15%)	
≥240, <460	14 (6%)	19 (11%)	4 (12%)	
≥460	43 (19%)	24 (14%)	5 (15%)	
Unknown, N (%)	1 (0.4%)	0 (0%)	0 (0%)	
***APOEe4* allele, N (%)**				0.155^a^
0	190 (83%)	125 (74%)	26 (79%)	
1	31 (14%)	35 (21%)	7 (21%)	
2	1 (0.4%)	3 (1.8%)	0 (0%)	
Unknown*	7 (3.1%)	5 (3.0%)	0 (0%)	
**Hypertension, N (%)**	94 (41%)	69 (41%)	11 (33%)	0.722^a^
**Diabetes, N (%)**	42 (18%)	22 (13%)	10 (30%)	0.050^a^
**Dyslipidemia, N (%)**	116 (51%)	75 (45%)	9 (27%)	0.034^a^

IQR, interquartile range. Unknowns were excluded from statistical analysis. Drinking index = amount of current alcohol consumption (g/day/60 kg body weight) × categorical years of habitual drinking (0–6).

*Participants with rs429358 T/C and rs7412 C/T (indistinguishable between *APOEε2/ε4* and *APOEε1/ε3*)

^a^Fisher’s exact test. ^b^Kruskal-Wallis test. ^c^Spearman’s rank correlation test.

### 3.2. Participants characteristics

As shown in Table 1, no significant differences were observed in sex, age, years of education, Brinkman index, and hypertension across *ALDH2* genotypes. The number of years of habitual drinking was negatively associated with the number of variant alleles (ρ = −0.16, P < 0.001). Although a distribution bias was observed in diabetes (P = 0.050), no trend was observed with variant allele numbers (ρ = −0.004, P = 0.92). Dyslipidemia showed a negative association with the rs671 variant (P = 0.034).

### 3.3. Neurological findings

**Fig. 1:**
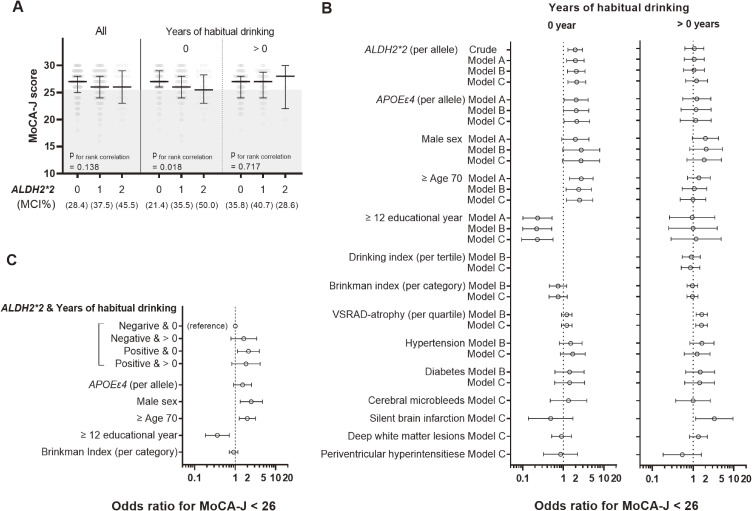
Association between *ALDH2* rs671 variant and mild cognitive impairment A) Distribution of MoCA-J scores by *ALDH2* rs671 genotypes for the total cohort (N = 430) and stratified cohorts by a history of habitual alcohol consumption, 0 year (N = 250) or >0 year (N = 175), where unknown drinking history was removed from (N = 5). Error bars indicate the median and interquartile range. The gray-shaded area represents MCI (MoCA-J < 26). The MCI rate is shown in the panel. B) Odds ratios for mild cognitive impairment defined by MoCA-J < 26 stratified by a history of habitual drinking. All covariates used in the modeling are shown. Akaike Information Criterion (smaller values indicate better model fit) in each model was 301, 263, 256, and 271 for Crude, Model A, Model B, and Model C, respectively, for the “0-year” stratum, and similarly, 235, 228, 225, and 225 for the “>0 years” stratum. C) Interaction between the rs671 variant and history of habitual drinking. Odds ratios were estimated using Model 2 in Table S3 with categorizations modified as indicated in the panel.

The MoCA-J scores tended to be lower in rs671 variant carriers (ρ = −0.07, P = 0.138) (Fig. 1A, left panel, “All”). MCI, defined by MoCA-J < 26, was more prevalent among variant carriers: 28.4% in ALDH2*1/*1, 37.5% in ALDH2*1/*2, and 45.5% in ALDH2*2/*2 (ρ = 0.12, P = 0.015). This trend was stronger in participants without a history of habitual alcohol consumption (ρ = −0.15, P = 0.018 for MoCA-J; ρ = 0.20, P = 0.001 for MCI) (Fig. 1A, center panel) and was not observed in those with a history of habitual drinking (ρ = 0.03, P = 0.717 for MoCA-J; ρ = 0.03, P = 0.704 for MCI) (Fig. 1A, right panel). The median (interquartile range) of VSRAD-atrophy values were below 1, 0.80 (0.49–1.00) (Table S2), indicating that 25% of participants had hippocampal atrophy exceeding age-related changes. Small vessel-related abnormalities included cerebral microbleeds in 13% (N = 56), DSWMH > Grade 0 in 44.7% (N = 192), and SBI > Grade 0 in 9.5% (N = 41), suggesting cerebral small vessel dysfunction.

### 3.4. Effects of *ALDH2* rs671 genotype on mild cognitive impairment

A positive association between the ALDH2*2 variant and MCI was consistently estimated using logistic regression models, including sensitivity analysis (Tables S3–S6). This association remained significant even after adjusting for habitual drinking, VSRAD-atrophy, and small vessel-related MRI findings, indicating that the association between rs671 and MCI is independent of these factors.

A marginal interaction between ALDH2*2 and years of habitual drinking was observed (P = 0.053) (Model 4 in Table S3). Table S7 and Fig. 1B show the stratified analysis results. A mild positive effect of the rs671 variant on MCI was consistently observed in participants without a history of habitual drinking, with odds ratios ranging from 1.9 to 2.1 after adjusting for sex, age, years of education, Brinkman index, hypertension, diabetes, and potential MRI findings. The rs671 variant was not a significant risk factor in participants with a history of habitual drinking, whereas hippocampal atrophy and subclinical stroke were positively associated with MCI. These associations were confirmed in sensitivity analyses (Tables S8–S10).

Figure 1C illustrates the interactive association by dividing participants into four groups based on the presence or absence of the ALDH2*2 variant and a history of habitual drinking. Point estimates suggested similar risks for the three groups, except for non-carriers of the rs671 variant without a history of habitual drinking.

A similar positive effect was estimated for *APOEε4* in participants without a history of habitual drinking (Table S7 and Fig. 1B). However, the expected interactive effect between ALDH2*2 and *APOEε4* was not observed (P = 0.270–0.506 when added to Models 2 to 4 in Table S3).

## 4. Discussion

We investigated the association between the *ALDH2* rs671 variant and MCI using data from the Brain MRI Checkup Cohort, consisting of Japanese residents who were independent in their activities of daily living and without apparent dementia. Our study is the first to demonstrate that the rs671 variant is a mild risk factor for MCI. This association persisted even after adjusting for habitual drinking, suggesting that the acetaldehyde-accumulating properties of rs671 may not be the primary mechanism involved. The association was independent of hippocampal atrophy and small vessel dysfunction as evaluated by MRI, which are well-established indicators of cognitive impairment [41–45]. This trend was more pronounced in individuals without a history of habitual alcohol use. The odds ratios for MCI were similar among participants with the rs671 variant, regardless of their drinking habits, and among those without the rs671 variant but with a drinking habit. These findings suggest that preventive interventions for dementia based on the rs671 variant may achieve better outcomes and point to an unrecognized biological mechanism for dementia development that is independent of hippocampal atrophy and small vessel disease.

Although ALDH2 is known for its role in alcohol metabolism, various phenotypes associated with the rs671 variant, independent of alcohol consumption, have been reported [46–48]. For example, the rs671 variant accelerates the progression of bone marrow failure in Fanconi anemia, a severe hereditary disorder characterized by defective DNA damage response and repair [49] due to endogenous aldehyde-derived DNA damage [50]. Additionally, the rs671 variant is associated with a higher susceptibility to coronary artery spasms, which is exacerbated by tobacco smoke [51–55]. These findings suggest that individuals with the rs671 variant should avoid exogenous aldehydes from sources such as heavy alcohol use, smoking, chronic stressors, and hyperglycemia [8, 9].

Given the high expression of ALDH2 in various species, including primates, algae, fish, and rodents [15], which are rarely exposed to ethanol, its fundamental role is likely the metabolism of endogenous aldehydes. The rs671 variant leads to the accumulation of endogenous aldehydes, such as 4-hydroxynonenal (4-HNE) and formaldehyde, due to reduced ALDH2 activity [16–18, 56–60]. This accumulation could explain the association between the rs671 variant and MCI through several mechanisms.

First, the failure of autophagy and lysosomal processes caused by the calpain-mediated cleavage of Hsp70.1, oxidized by 4-HNE, leads to neuronal death [21]. This process results in the aggregation of amyloid β (Aβ) peptide and α-synuclein [61], with increased 4-HNE levels in the amygdala, hippocampus, and parahippocampal gyrus of Alzheimer’s disease patients [62]. The association between the rs671 variant and increased 4-HNE in the hippocampus [63] and the alleviation of cognitive decline in rs671 animal models through the suppression of diet-derived aldehyde production [64] support this theory.

Second, the over-accumulation of formaldehyde causes structural changes in N-Methyl-D-Aspartate (NMDA) receptors, which are involved in learning and memory, thereby impairing memory [19]. Given that formaldehyde is a substrate of ALDH2, the association between urinary formaldehyde concentration and cognitive decline [19, 65] suggests a reasonable link between the rs671 variant and MCI. Formaldehyde is abundant *in vivo* because of the demethylation of various organic compounds, such as methylated DNA [66], metabolites of folic acid in the one-carbon cycle [67], and sarcosine in neurons [19].

Our study is the first to demonstrate that the *ALDH2* rs671 variant is associated with MCI, independent of alcohol consumption, hippocampal atrophy, and small vessel dysfunction. However, this study has several limitations that need to be addressed. First, the accuracy of diagnosis is limited. While the Montreal Cognitive Assessment (MoCA) is a well-established tool frequently used for assessing MCI and Alzheimer’s disease [68], its accuracy for distinguishing MCI in healthy older individuals ranges from 0.87 to 0.99 for the area under the ROC curve [69]. Although a cutoff value of 26 is commonly recommended [33], some studies suggest using a cutoff of MoCA < 25 (sensitivity 80.5%, specificity 81.2%) for individuals aged 60 years or above [40]. We confirmed the validity of both cutoffs in our primary and sensitivity analyses. Second, the accuracy of lifetime alcohol consumption data, which is strongly influenced by the rs671 variant and impacts dementia risk [70], was limited. This limitation may have hindered our ability to detect interactions between rs671 and alcohol use, which is crucial information for preventive medicine. Third, the sample size was insufficient to replicate the synergistic effect between *APOEε4* and the rs671 variant demonstrated in previous studies [25]. The *APOEε4* carrier rate in our study was 18.4%, similar to that in previous reports [71], but insufficient for verifying this interaction. We had only four participants with *APOEε4/ε4* (0.9%), whereas the aforementioned case-control study included 37 *APOEε4/ε4* cases [25]. Finally, the scientific reliability of these findings is constrained by the underlying molecular mechanisms being unknown and the limited size of the study. Further evidence is required before considering clinical application.

## 5. Conclusion

For the first time, our study findings suggest that the *ALDH2* rs671 variant is a mild risk factor for MCI, independent of alcohol use, hippocampal atrophy, and cerebral small vessel dysfunction, using a cross-sectional approach with a Japanese Brain MRI Checkup Cohort database. This alcohol-independent association suggests a possible mechanism involving the accumulation of endogenous aldehydes due to the rs671 variant. Our findings suggest the need for race-specific preventive approaches for MCI based on the rs671 genotype and highlight an unidentified mechanism for the development of cognitive decline.

## Abbreviations

ALDH2: aldehyde dehydrogenase 2
MCI: mild cognitive impairment
MoCA: Montreal Cognitive Assessment
VSRAD: Voxel-based Specific Regional Analysis System for Atrophy Detection
SBI: silent brain infarction
DSWMH: deep and subcortical white matter hyperintensity

## Supplementary information

**Additional file 1:** **Figure S1.** Histograms of VSRAD-atrophy. **Table S1.** Genotyping result for *APOE*. **Table S2.** Magnetic resonance imaging (MRI) findings. **Table S3.** Logistic regression analysis for mild cognitive impairment, MoCA < 26. **Table S4.** Sensitivity analysis 1: logistic regression for mild cognitive impairment (MoCA < 26). **Table S5.** Sensitivity analysis 2: logistic regression for mild cognitive impairment (MoCA < 26). **Table S6.** Sensitivity analysis 3: logistic regression with a different definition for MCI, MoCA < 25. **Table S7.** Logistic regression analysis for mild cognitive impairment (MoCA < 26) by history of habitual drinking. **Table S8.** Sensitivity analysis 4: logistic regression for mild cognitive impairment (MoCA < 25) by history of habitual drinking. **Table S9.** Sensitivity analysis 5: logistic regression for mild cognitive impairment (MoCA < 26) in participants with a history of habitual drinking. **Table S10.** Sensitivity analysis 6: logistic regression for mild cognitive impairment (MoCA < 26) in participants with a history of habitual drinking.

## Declarations

### Availability of data and materials

The datasets in the current study are not publicly available to protect the participants’ privacy, but they are available from the corresponding author upon reasonable request.
