# Supplementary material for: Asian flush gene variant increases mild cognitive impairment risk: a cross-sectional study of the Yoshinogari Brain MRI Checkup Cohort
Source: Environ Health Prev Med. 2024 Oct 11;29:55. doi: 10.1265/ehpm.24-00214 (PMC11473384; doi:10.1265/ehpm.24-00214)
Supplement: Supplementary file 1 — Additional file 1: Figure S1. Histograms of VSRAD-atrophy. Table S1. Genotyping result for APOE. Table S2. Magnetic resonance imaging (MRI) findings. Table S3. Logistic regression analysis for mild cognitive impairment, MoCA < 26. Table S4. Sensitivity analysis 1: logistic regression for mild cognitive impairment (MoCA < 26). Table S5. Sensitivity analysis 2: logistic regression for mild cognitive impairment (MoCA < 26). Table S6. Sensitivity analysis 3: logistic regression with a different definition for MCI, MoCA < 25. Table S7. Logistic regression analysis for mild cognitive impairment (MoCA < 26) by history of habitual drinking. Table S8. Sensitivity analysis 4: logistic regression for mild cognitive impairment (MoCA < 25) by history of habitual drinking. Table S9. Sensitivity analysis 5: logistic regression for mild cognitive impairment (MoCA < 26) in participants with a history of habitual drinking. Table S10. Sensitivity analysis 6: logistic regression for mild cognitive impairment (MoCA < 26) in participants with a history of habitual drinking. [file ehpm-29-055-s001.docx]

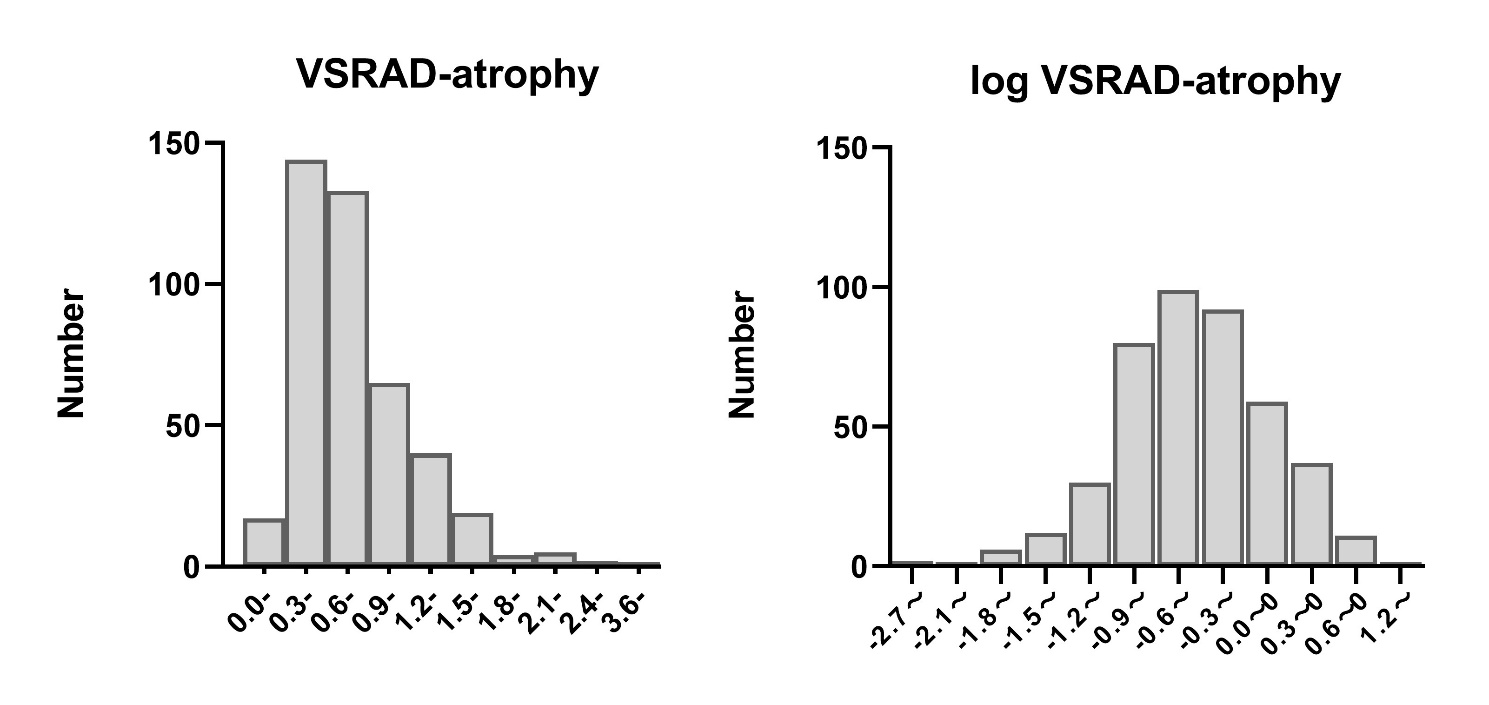


**Figure S1. Histograms of VSRAD-atrophy.**

**Table S1. Genotyping result for *APOE.***

| rs429358 | rs7412 | Genotype | N | % |
| --- | --- | --- | --- | --- |
| TT | TT | *APOEε2ε2* | 1 | 0.23 |
| TT | CT | *APOEε2ε3* | 31 | 7.21 |
| TC | CT | *APOEε1ε3* or *APOEε2ε4* | 6 | 1.40 |
| TT | CC | *APOEε3ε3* | 309 | 71.86 |
| TC | CC | *APOEε3ε4* | 73 | 16.98 |
| CC | CC | *APOEε4ε4* | 4 | 0.93 |
| CC | CT | *APOEε1ε4* | 0 | 0.00 |
| CC | TT | *APOEε1ε1* | 0 | 0.00 |
| TT | FALSE | Unknown | 2 | 0.47 |
| FALSE | CC | Unknown | 3 | 0.70 |
| FALSE | CT | Unknown | 1 | 0.23 |
| FALSE, analytical failure in real-time PCR | | | | |

| **Table S2. Magnetic resonance imaging (MRI) findings.** | | |
| --- | --- | --- |
| **VSRAD-atrophy** | Median (IQR) | 0.80 (0.49-1.00) |
| **Log (VSRAD-atrophy)** | Mean ± SD | -0.35 ± 0.53 |
|  |  |  |
| **Cerebral microbleeds** | N (%) | 56 (13.0%) |
| **Silent brain infarction** | N (%) | 41 (9.5%) |
| **Deep white matter lesions*** |  |  |
| Grade 0 | N (%) | 238 (55.3%) |
| Grade 1 |  | 188 (43.7%) |
| Grade 2 |  | 4 (0.9%) |
| Grade 3 |  | 0 (0%) |
| **Periventricular hyperintensities*** |  |  |
| Grade 0 | N (%) | 378 (87.9%) |
| Grade 1 |  | 47 (10.9%) |
| Grade 2 |  | 5 (1.2%) |
| Grade 3 |  | 0 (0%) |
| *Graded using the Fazekas scale. IQR, interquartile range. SD, standard deviation.  **Deep white matter lesions** and **Periventricular hyperintensities**: Grade 0 = T2 high signal lesions was graded in absent, Grade 1 = punctate foci; Grade 2 = beginning confluence of foci, Grade 3 = large fused areas | | |

| **Table S3. Logistic regression analysis for mild cognitive impairment, MoCA <26.** | | | | | | | | | | | | | | |
| --- | --- | --- | --- | --- | --- | --- | --- | --- | --- | --- | --- | --- | --- | --- |
|  | Crude | | Model 1 | | Model 2 | | Model 3 | | Model 4 | | Model 5 | | |  |
|  | AIC = 545 | | AIC = 526 | | AIC = 492 | | AIC = 478 | | AIC = 476 | | AIC = 481 | | |  |
|  | N = 430 | | N = 418 | | N = 418 | | N = 412 | | N = 412 | | N = 412 | | |  |
|  | β | *p* | β | *p* | β | *p* | β | *p* | β | *p* | β | *p* |  |  |
| Intercept | -0.92 | < 0.001 | -1.03 | < 0.001 | -5.69 | < 0.001 | -3.86 | 0.030 | -3.65 | 0.041 | -3.40 | 0.061 |  |  |
| **Number of *ALDH2*2*** | 0.39 | 0.015 | 0.38 | 0.018 | 0.37 | 0.032 | 0.40 | 0.033 | 0.65 | 0.004 | 0.66 | 0.004 |  |  |
| **Number of *APOEε4*** |  |  | 0.54 | 0.025 | 0.46 | 0.072 | 0.36 | 0.182 | 0.39 | 0.155 | 0.41 | 0.142 |  |  |
| Male sex |  |  |  |  | 0.88 | < 0.001 | 0.99 | 0.003 | 0.95 | 0.005 | 0.90 | 0.009 |  |  |
| Age |  |  |  |  | 0.07 | 0.001 | 0.05 | 0.038 | 0.04 | 0.063 | 0.04 | 0.098 |  |  |
| Years of education, ≥ 12 |  |  |  |  | -0.98 | 0.004 | -1.04 | 0.003 | -1.08 | 0.002 | -1.05 | 0.003 |  |  |
| Years of habitual drinking (per category) |  |  |  |  |  |  | -0.03 | 0.635 | 0.07 | 0.453 | 0.06 | 0.472 |  |  |
| Brinkmann index (per category) |  |  |  |  |  |  | -0.06 | 0.625 | -0.09 | 0.510 | -0.10 | 0.473 |  |  |
| log (VSRAD-atrophy) |  |  |  |  |  |  | 0.82 | < 0.001 | 0.85 | < 0.001 | 0.85 | < 0.001 |  |  |
| Hypertension |  |  |  |  |  |  | 0.50 | 0.034 | 0.53 | 0.026 | 0.46 | 0.067 |  |  |
| Diabetes |  |  |  |  |  |  | 0.34 | 0.235 | 0.32 | 0.274 | 0.31 | 0.292 |  |  |
| *ALDH2*2* × years of habitual drinking |  |  |  |  |  |  |  |  | -0.18 | 0.053 | -0.18 | 0.058 |  |  |
| Cerebral microbleeds |  |  |  |  |  |  |  |  |  |  | 0.16 | 0.642 |  |  |
| Silent brain infarction |  |  |  |  |  |  |  |  |  |  | 0.31 | 0.430 |  |  |
| Deep white matter lesions |  |  |  |  |  |  |  |  |  |  | 0.08 | 0.669 |  |  |
| Periventricular hyperintensities |  |  |  |  |  |  |  |  |  |  | -0.34 | 0.345 |  |  |
| Years of habitual drinking and Brinkmann index were categorized into 7 and 4, respectively, as shown in Table 1.  AIC: Akaike information criterion (smaller values indicate better model fit). β, partial regression coefficient | | | | | | | | | | | | | | |

| **Table S4. Sensitivity analysis 1: logistic regression for mild cognitive impairment (MoCA < 26).** | | | | | | |
| --- | --- | --- | --- | --- | --- | --- |
|  | Model 3 | | Model 4 | | Model 5 | |
|  | AIC = 480 | | AIC = 481 | | AIC = 487 | |
|  | N = 417 | | N = 417 | | N = 417 | |
|  | β | *p* | β | *p* | β | *p* |
| Intercept | -4.66 | 0.011 | -4.61 | 0.012 | -4.33 | 0.021 |
| **Number of *ALDH2*2*** | 0.49 | 0.007 | 0.54 | 0.007 | 0.56 | 0.006 |
| Number of *APOEε4* | 0.45 | 0.099 | 0.45 | 0.099 | 0.46 | 0.092 |
| Male sex | 0.78 | 0.015 | 0.77 | 0.015 | 0.71 | 0.026 |
| Age | 0.06 | 0.015 | 0.06 | 0.017 | 0.05 | 0.032 |
| Years of education ≥ 12 | -1.08 | 0.002 | -1.09 | 0.002 | -1.07 | 0.003 |
| Current alcohol consumption (10 g/day/60 kg body weight) | 0.13 | 0.094 | 0.16 | 0.087 | 0.17 | 0.081 |
| Brinkmann index (per category) | -0.11 | 0.389 | -0.12 | 0.365 | -0.13 | 0.327 |
| log (VSRAD-atrophy) | 0.79 | 0.001 | 0.80 | 0.001 | 0.80 | 0.001 |
| Hypertension | 0.44 | 0.062 | 0.45 | 0.056 | 0.37 | 0.132 |
| Diabetes | 0.41 | 0.158 | 0.39 | 0.171 | 0.38 | 0.191 |
| *ALDH2*2 ×* current alcohol consumption |  |  | -0.01 | 0.569 | -0.01 | 0.578 |
| Cerebral microbleeds |  |  |  |  | 0.09 | 0.800 |
| Silent brain infarction |  |  |  |  | 0.38 | 0.333 |
| Deep white matter lesions (per category) |  |  |  |  | 0.10 | 0.598 |
| Periventricular hyperintensities (per category) |  |  |  |  | -0.34 | 0.332 |
| The covariate “years of habitual drinking” in Table S3 was replaced by “current alcohol consumption.”  AIC: Akaike information criterion (smaller values indicate better model fit). β, partial regression coefficient | | | | | | |

| **Table S5. Sensitivity analysis 2: logistic regression for mild cognitive impairment (MoCA < 26).** | | | | | | |
| --- | --- | --- | --- | --- | --- | --- |
|  | Model 3 | | Model 4 | | Model 5 | |
|  | AIC = 478 | | AIC = 479 | | AIC = 485 | |
|  | N = 412 | | N = 412 | | N = 412 | |
|  | β | *p* | β | *p* | β | *p* |
| Intercept | -4.02 | 0.026 | -3.97 | 0.028 | -3.72 | 0.044 |
| **Number of *ALDH2*2*** | 0.44 | 0.017 | 0.47 | 0.018 | 0.49 | 0.014 |
| Number of *APOEε4* | 0.37 | 0.172 | 0.38 | 0.168 | 0.39 | 0.159 |
| Male sex | 0.92 | 0.004 | 0.91 | 0.004 | 0.85 | 0.008 |
| Age | 0.05 | 0.034 | 0.05 | 0.038 | 0.05 | 0.063 |
| Years of education ≥ 12 | -1.06 | 0.003 | -1.07 | 0.002 | -1.04 | 0.003 |
| Drinking index | 0.01 | 0.688 | 0.13 | 0.556 | 0.14 | 0.529 |
| Brinkmann index (per category) | -0.10 | 0.479 | -0.10 | 0.454 | -0.11 | 0.405 |
| log (VSRAD-atrophy) | 0.81 | 0.001 | 0.82 | 0.001 | 0.81 | 0.001 |
| Hypertension | 0.48 | 0.042 | 0.49 | 0.039 | 0.41 | 0.097 |
| Diabetes | 0.36 | 0.209 | 0.36 | 0.219 | 0.34 | 0.236 |
| *ALDH2*2 ×* drinking index |  |  | -0.01 | 0.661 | -0.01 | 0.688 |
| Cerebral microbleeds |  |  |  |  | 0.11 | 0.747 |
| Silent brain infarction |  |  |  |  | 0.37 | 0.348 |
| Deep white matter lesions |  |  |  |  | 0.08 | 0.660 |
| Periventricular hyperintensities |  |  |  |  | -0.34 | 0.339 |
| The covariate "years of habitual drinking" in Table S3 was replaced by "drinking index."  Drinking index = current alcohol consumption (10 g/day/60 kg body weight) × categorical years of habitual drinking*  *0 = “never”, 1 = “< 10 y”, 2 = “10–19 y”, 3 = “20–29 y”, 4 = “30–39 y”, 5 = “40–49 y“, and 6 = “≥50 y”  AIC: Akaike information criterion (smaller values indicate better model fit). β, partial regression coefficient | | | | | | |

| **Table S6. Sensitivity analysis 3: logistic regression with a different definition for MCI, MoCA < 25.** | | | | | | | | | | | | | |
| --- | --- | --- | --- | --- | --- | --- | --- | --- | --- | --- | --- | --- | --- |
|  | Crude | | Model 1 | | Model 2 | | Model 3 | | Model 4 | | Model 5 | |  |
|  | AIC = 487 | | AIC = 469 | | AIC = 444 | | AIC = 431 | | AIC = 431 | | AIC = 437 | |  |
|  | N = 430 | | N = 418 | | N = 418 | | N = 412 | | N = 412 | | N = 412 | |  |
|  | β | *p* | β | *p* | β | *p* | β | *p* | β | *p* | β | *p* |  |
| Intercept | -1.31 | < 0.001 | -1.45 | < 0.001 | -6.95 | < 0.001 | -5.14 | 0.008 | -4.92 | 0.012 | -4.62 | 0.020 |  |
| **Number of *ALDH2*2*** | 0.42 | 0.014 | 0.40 | 0.020 | 0.38 | 0.037 | 0.38 | 0.053 | 0.59 | 0.014 | 0.59 | 0.015 |  |
| **Number of *APOEε4*** |  |  | 0.73 | 0.004 | 0.67 | 0.011 | 0.57 | 0.041 | 0.60 | 0.033 | 0.61 | 0.030 |  |
| Male sex |  |  |  |  | 0.74 | 0.002 | 0.74 | 0.038 | 0.71 | 0.047 | 0.69 | 0.056 |  |
| Age |  |  |  |  | 0.08 | 0.001 | 0.06 | 0.018 | 0.06 | 0.028 | 0.05 | 0.053 |  |
| Years of education ≥ 12 |  |  |  |  | -0.75 | 0.028 | -0.80 | 0.025 | -0.84 | 0.020 | -0.80 | 0.029 |  |
| Years of habitual drinking (per category) |  |  |  |  |  |  | -0.06 | 0.462 | 0.03 | 0.764 | 0.02 | 0.832 |  |
| Brinkmann index (per category) |  |  |  |  |  |  | 0.05 | 0.748 | 0.02 | 0.861 | 0.02 | 0.909 |  |
| log (VSRAD-atrophy) |  |  |  |  |  |  | 0.86 | < 0.001 | 0.90 | < 0.001 | 0.90 | < 0.001 |  |
| Hypertension |  |  |  |  |  |  | -0.05 | 0.836 | -0.03 | 0.903 | -0.16 | 0.554 |  |
| Diabetes |  |  |  |  |  |  | 0.52 | 0.084 | 0.49 | 0.102 | 0.49 | 0.102 |  |
| *ALDH2*2* × years of habitual drinking |  |  |  |  |  |  |  |  | -0.15 | 0.133 | -0.15 | 0.145 |  |
| Cerebral microbleeds |  |  |  |  |  |  |  |  |  |  | 0.32 | 0.386 |  |
| Silent brain infarction |  |  |  |  |  |  |  |  |  |  | 0.31 | 0.439 |  |
| Deep white matter lesions |  |  |  |  |  |  |  |  |  |  | 0.10 | 0.630 |  |
| Periventricular hyperintensities |  |  |  |  |  |  |  |  |  |  | -0.08 | 0.821 |  |
| The same covariates were included in Table S3  AIC: Akaike information criterion (smaller values indicate better model fit). β, partial regression coefficient.  ~~.~~ | | | | | | | | | | | | | |

| **Table S7. Logistic regression analysis for mild cognitive impairment (MoCA < 26) by history of habitual drinking** | | | | | | | | | | | | | |  |  |
| --- | --- | --- | --- | --- | --- | --- | --- | --- | --- | --- | --- | --- | --- | --- | --- |
|  | **Stratified Analysis: Year of habitual drinking (0 years)** | | | | | | | | | | | | |  |  |
|  | Crude | | | Mode a | | | Model b | | Model c | | Model d | | |  |  |
|  | AIC = 301 | | | AIC = 292 | | | AIC = 258 | | AIC = 259 | | AIC = 265 | | |  |  |
|  | N = 250 | | | N = 246 | | | N = 246 | | N = 245 | | N = 245 | | |  |  |
|  | β | *p* | β | | *p* | β | | *p* | β | *p* | | β | *p* | |  |
| Intercept | -1.29 | <0.001 | -1.44 | | <0.001 | -9.24 | | <0.001 | -8.30 | 0.003 | | -9.13 | 0.002 | |  |
| Number of *ALDH2*2* | 0.67 | 0.001 | 0.67 | | 0.002 | 0.65 | | 0.012 | 0.69 | 0.008 | | 0.72 | 0.007 | |  |
| Number of *APOEε4* |  |  | 0.67 | | 0.037 | 0.74 | | 0.035 | 0.75 | 0.040 | | 0.79 | 0.036 | |  |
| Male sex |  |  |  | |  | 0.69 | | 0.085 | 1.03 | 0.062 | | 1.01 | 0.069 | |  |
| Age |  |  |  | |  | 0.12 | | <0.001 | 0.11 | 0.003 | | 0.12 | 0.002 | |  |
| Years of education ≥12 |  |  |  | |  | -1.36 | | 0.002 | -1.39 | 0.002 | | -1.35 | 0.003 | |  |
| Brinkmann Index (per category) |  |  |  | |  |  | |  | -0.30 | 0.245 | | -0.28 | 0.290 | |  |
| log (VSRAD -atrophy) |  |  |  | |  |  | |  | 0.41 | 0.217 | | 0.40 | 0.228 | |  |
| Hypertension |  |  |  | |  |  | |  | 0.36 | 0.281 | | 0.51 | 0.160 | |  |
| Diabetes |  |  |  | |  |  | |  | 0.38 | 0.369 | | 0.40 | 0.362 | |  |
| Cerebral microbleeds |  |  |  | |  |  | |  |  |  | | 0.23 | 0.667 | |  |
| Silent brain infarction |  |  |  | |  |  | |  |  |  | | -0.86 | 0.193 | |  |
| Deep white matter lesions |  |  |  | |  |  | |  |  |  | | -0.16 | 0.588 | |  |
| Periventricular hyperintensities |  |  |  | |  |  | |  |  |  | | -0.12 | 0.807 | |  |
|  | **Stratified Analysis: Year of habitual drinking (> 0 years)** | | | | | | | | | | | | | | |
|  | Crude | | Model a | | | Model b | | | Model c | | | Model d | | | |
|  | AIC = 235 | | AIC = 227 | | | AIC = 228 | | | AIC = 218 | | | AIC = 217 | | | |
|  | N = 175 | | N = 167 | | | N = 167 | | | N = 167 | | | N = 167 | | | |
|  | β | p | β | | p | β | | p | β | p | | β | p | | |
| Intercept | -0.55 | 0.004 | -0.58 | | 0.006 | -2.63 | | 0.252 | -0.63 | 0.804 | | 0.07 | 0.978 | | |
| Number of *ALDH2*2* | 0.07 | 0.807 | 0.07 | | 0.791 | 0.08 | | 0.788 | 0.03 | 0.921 | | 0.20 | 0.527 | | |
| Number of *APOEε4* |  |  | 0.22 | | 0.576 | 0.20 | | 0.618 | 0.23 | 0.606 | | 0.24 | 0.601 | | |
| Male sex |  |  |  | |  | 0.70 | | 0.060 | 0.81 | 0.087 | | 0.71 | 0.149 | | |
| Age |  |  |  | |  | 0.02 | | 0.446 | -0.01 | 0.833 | | -0.02 | 0.536 | | |
| Years of education ≥12 |  |  |  | |  | -0.06 | | 0.921 | 0.09 | 0.897 | | 0.30 | 0.677 | | |
| Brinkmann Index (per category) |  |  |  | |  |  | |  | -0.06 | 0.703 | | -0.07 | 0.685 | | |
| log (VSRAD -atrophy) |  |  |  | |  |  | |  | 1.41 | <0.001 | | 1.42 | <0.001 | | |
| Hypertension |  |  |  | |  |  | |  | 0.51 | 0.145 | | 0.20 | 0.602 | | |
| Diabetes |  |  |  | |  |  | |  | 0.56 | 0.183 | | 0.52 | 0.230 | | |
| Cerebral microbleeds |  |  |  | |  |  | |  |  |  | | -0.05 | 0.918 | | |
| Silent brain infarction |  |  |  | |  |  | |  |  |  | | 1.38 | 0.016 | | |
| Deep white matter lesions |  |  |  | |  |  | |  |  |  | | 0.29 | 0.278 | | |
| Periventricular hyperintensities |  |  |  | |  |  | |  |  |  | | -0.59 | 0.304 | | |
| AIC: Akaike Information Criterion (smaller values indicate better model fit). β, partial regression coefficients. | | | | | | | | | | | | | | | |

| **Table S8. Sensitivity analysis 4: logistic regression for mild cognitive impairment (MoCA < 25) by history of habitual drinking** | | | | | | | | | | |  |
| --- | --- | --- | --- | --- | --- | --- | --- | --- | --- | --- | --- |
|  | **Stratified Analysis: Year of habitual drinking (0 years)** | | | | | | | | | |  |
|  | Crude | | Mode a | | Model b | | Model c | | Model d | |  |
|  | AIC = 268 | | AIC = 260 | | AIC = 239 | | AIC = 239 | | AIC = 244 | |  |
|  | N = 250 | | N = 246 | | N = 246 | | N = 245 | | N = 245 | |  |
|  | β | *p* | β | *p* | β | *p* | β | *p* | β | *p* |  |
| Intercept | -1.64 | <0.001 | -1.82 | <0.001 | -9.75 | <0.001 | -9.66 | 0.001 | -10.76 | <0.001 |  |
| Number of *ALDH2*2* | 0.66 | 0.003 | 0.68 | 0.003 | 0.58 | 0.028 | 0.63 | 0.020 | 0.61 | 0.029 |  |
| Number of *APOEε4* |  |  | 0.74 | 0.024 | 0.78 | 0.027 | 0.83 | 0.028 | 0.85 | 0.027 |  |
| Male sex |  |  |  |  | 0.71 | 0.078 | 0.93 | 0.090 | 0.95 | 0.084 |  |
| Age |  |  |  |  | 0.12 | <0.001 | 0.12 | 0.003 | 0.13 | 0.002 |  |
| Years of education ≥12 |  |  |  |  | -0.90 | 0.037 | -0.91 | 0.045 | -0.74 | 0.122 |  |
| Brinkmann Index (per category) |  |  |  |  |  |  | -0.29 | 0.278 | -0.25 | 0.364 |  |
| log (VSRAD -atrophy) |  |  |  |  |  |  | 0.33 | 0.341 | 0.29 | 0.402 |  |
| Hypertension |  |  |  |  |  |  | -0.10 | 0.772 | -0.08 | 0.838 |  |
| Diabetes |  |  |  |  |  |  | 0.80 | 0.064 | 0.86 | 0.053 |  |
| Cerebral microbleeds |  |  |  |  |  |  |  |  | 0.72 | 0.184 |  |
| Silent brain infarction |  |  |  |  |  |  |  |  | -0.59 | 0.386 |  |
| Deep white matter lesions |  |  |  |  |  |  |  |  | -0.30 | 0.338 |  |
| Periventricular hyperintensities |  |  |  |  |  |  |  |  | 0.37 | 0.470 |  |
|  | **Stratified Analysis: Year of habitual drinking (>0 years)** | | | | | | | | | |  |
|  | Crude | | Model a | | Model b | | Model c | | Model d | |  |
|  | AIC = 211 | | AIC = 204 | | AIC = 207 | | AIC = 197 | | AIC = 198 | |  |
|  | N = 175 | | N = 167 | | N = 167 | | N = 167 | | N = 167 | |  |
|  | β | p | β | p | β | p | β | p | β | p | |
| Intercept | -1.00 | <0.001 | -1.06 | <0.001 | -4.06 | 0.102 | -1.59 | 0.563 | -0.91 | 0.750 | |
| Number of *ALDH2*2* | 0.13 | 0.644 | 0.09 | 0.768 | 0.12 | 0.695 | 0.08 | 0.801 | 0.25 | 0.464 | |
| Number of *APOEε4* |  |  | 0.55 | 0.165 | 0.48 | 0.253 | 0.53 | 0.238 | 0.55 | 0.235 | |
| Male sex |  |  |  |  | 0.45 | 0.259 | 0.45 | 0.374 | 0.35 | 0.504 | |
| Age |  |  |  |  | 0.04 | 0.193 | 0.01 | 0.809 | 0.00 | 0.908 | |
| Years of education ≥12 |  |  |  |  | -0.34 | 0.606 | -0.20 | 0.780 | -0.08 | 0.909 | |
| Brinkmann Index (per category) |  |  |  |  |  |  | 0.08 | 0.647 | 0.07 | 0.689 | |
| log (VSRAD -atrophy) |  |  |  |  |  |  | 1.52 | <0.001 | 1.55 | <0.001 | |
| Hypertension |  |  |  |  |  |  | -0.15 | 0.694 | -0.52 | 0.216 | |
| Diabetes |  |  |  |  |  |  | 0.50 | 0.259 | 0.47 | 0.300 | |
| Cerebral microbleeds |  |  |  |  |  |  |  |  | -0.15 | 0.783 | |
| Silent brain infarction |  |  |  |  |  |  |  |  | 1.21 | 0.032 | |
| Deep white matter lesions |  |  |  |  |  |  |  |  | 0.32 | 0.250 | |
| Periventricular hyperintensities |  |  |  |  |  |  |  |  | -0.60 | 0.318 | |
| The same covariates were included in Table S7  AIC: Akaike Information Criterion (smaller values indicate better model fit). β, partial regression coefficients | | | | | | | | | | | |

| **Table S9. Sensitivity analysis 5: logistic regression for mild cognitive impairment (MoCA < 26)** **in participants with a history of habitual drinking.** | | | | |
| --- | --- | --- | --- | --- |
|  | Model c | | Model d | |
|  | AIC = 220 | | AIC = 218 | |
|  | N = 167 | | N = 167 | |
|  | β | *p* | β | *p* |
| Intercept | -0.26 | 0.921 | 0.45 | 0.868 |
| Number of *ALDH2*2* | 0.02 | 0.938 | 0.20 | 0.539 |
| Number of *APOEε4* | 0.20 | 0.657 | 0.21 | 0.647 |
| Male sex | 0.85 | 0.078 | 0.77 | 0.131 |
| Age | -0.01 | 0.794 | -0.02 | 0.506 |
| Years of education ≥12 | 0.02 | 0.975 | 0.23 | 0.757 |
| Current alcohol consumption (10 g/day/60 kg body weight) | -0.12 | 0.652 | -0.14 | 0.614 |
| Brinkmann Index (per category) | -0.05 | 0.731 | -0.06 | 0.713 |
| log (VSRAD -atrophy) | 1.40 | 0.000 | 1.41 | 0.000 |
| Hypertension | 0.49 | 0.163 | 0.18 | 0.640 |
| Diabetes | 0.53 | 0.213 | 0.49 | 0.269 |
| Cerebral microbleeds |  |  | -0.05 | 0.921 |
| Silent brain infarction |  |  | 1.39 | 0.015 |
| Deep white matter lesions |  |  | 0.28 | 0.289 |
| Periventricular hyperintensities |  |  | -0.57 | 0.320 |
| "Current alcohol consumption" was added to the covariates in Table S7 (under the table). AIC: Akaike information criterion (smaller values indicate better model fit). β, partial regression coefficient. | | | | |

| **Table S10. Sensitivity analysis 6: logistic regression for mild cognitive impairment (MoCA < 26)** **in participants with a history of habitual drinking.** | | | | |
| --- | --- | --- | --- | --- |
|  | Model c | | Model d | |
|  | AIC = 220 | | AIC = 218 | |
|  | N = 167 | | N = 167 | |
|  | β | p | β | p |
| Intercept | -0.32 | 0.905 | 0.50 | 0.856 |
| Number of *ALDH2*2* | 0.03 | 0.932 | 0.20 | 0.536 |
| Number of *APOEε4* | 0.21 | 0.644 | 0.21 | 0.642 |
| Male sex | 0.84 | 0.081 | 0.77 | 0.131 |
| Age | -0.01 | 0.797 | -0.02 | 0.496 |
| Years of education ≥12 | 0.04 | 0.953 | 0.24 | 0.749 |
| Drinking index | -0.09 | 0.731 | -0.14 | 0.620 |
| Brinkmann Index (per category) | -0.06 | 0.714 | -0.06 | 0.701 |
| log (VSRAD -atrophy) | 1.40 | 0.000 | 1.41 | 0.000 |
| Hypertension | 0.50 | 0.161 | 0.17 | 0.648 |
| Diabetes | 0.54 | 0.209 | 0.49 | 0.273 |
| Cerebral microbleeds |  |  | -0.05 | 0.922 |
| Silent brain infarction |  |  | 1.39 | 0.015 |
| Deep white matter lesions |  |  | 0.28 | 0.281 |
| Periventricular hyperintensities |  |  | -0.57 | 0.312 |
| "Drinking Index" was added to the covariates in Table S7 (under the table). Drinking index = current alcohol consumption (10 g/day/60 kg body weight) × categorical years of habitual drinking*  *0 = “never,” 1 = “< 10 y,” 2 = “10–19 y,” 3 = “20–29 y,” 4 = “30–39 y,” 5 = “40–49 y," and 6 = “≥50 y”  AIC: Akaike Information Criterion (smaller values indicate better model fit). β, partial regression coefficients | | | | |
